# An Unusual Case of Bony Styloid Processes That Extend to the Hyoid Bone

**DOI:** 10.1155/2015/780870

**Published:** 2015-06-22

**Authors:** Shekhar K. Gadkaree, Christopher G. Hyppolite, Aisha Harun, Ryan H. Sobel, Young Kim

**Affiliations:** ^1^Department of Otolaryngology-Head & Neck Surgery, Johns Hopkins University School of Medicine, Baltimore, MD 21287, USA; ^2^Indiana University School of Medicine, Indianapolis, IN 46202, USA; ^3^Department of Surgery, Inova Fairfax Hospital, Virginia Commonwealth University School of Medicine, Inova Campus, Falls Church, VA 22042, USA

## Abstract

The embryological origin of the hyoid bone is a point of uncertainty, with controversy surrounding the relative contribution of the second pharyngeal arch to hyoid development. We encountered a 52-year-old male with bilateral bony styloid extension to the lesser cornu of the hyoid bone during the workup of a patient with laryngeal cancer. This embryological malformation clearly supports the hypothesis that the second pharyngeal arch gives rise to the lesser cornu and demonstrates an unusual clinical finding that may be encountered by otolaryngologists. We demonstrate the imaging findings and surgical management of this unusual anatomical variant and review the embryological basis for this rare malformation.

## 1. Introduction

The hyoid is a free floating horseshoe-shaped bone that sits in the anterior midline and serves several functions to assist in swallowing and movement on the tongue. It also serves as an attachment point for muscles of the pharynx and larynx, including the middle pharyngeal constrictor, hyoglossal, digastric, stylohyoid, geniohyoid, and mylohyoid muscles superiorly and the thyrohyoid, omohyoid, and sternohyoid muscles inferiorly [1]. These muscles serve as anchors for the hyoid bone as it has no bony attachments.

There are two embryologic hypotheses regarding derivation of the hyoid bone. The first suggests that hyoid bone is derived from the second and third pharyngeal arches. Evidence from embryological studies examining development patterns of the hyoid, such as the observation of unique fibrous tissue populations between the lesser cornu and hyoid body thereby implying the fusion of cells from two separate origins, has led to the greater acceptance of the theory involving the contribution of the second pharyngeal arch to hyoid bone development [2, 3]. The clinical entity of calcified styloid ligaments supports this contribution of Reichart's cartilage in the generation of the hyoid bone [4]. A second, alternate hypothesis states that the hyoid bone and all of its component parts originated from the third pharyngeal arch, which calls into question the notion that the second pharyngeal arch, also called Reichert's Cartilage, plays any role in development [3, 5]. While studies have examined the relative anatomic variation present in hyoid bone development, opportunities to examine these anomalies, particularly in adults without a history of previous surgery or trauma to the hyoid area, are poorly described in existing literature [6].

Knowledge of hyoid bone anatomy becomes important during surgical cases of trauma, pediatric otolaryngology, and laryngology where the anatomic relationships between the hyoid bone and its muscular attachments are critical [7]. In surgical oncology, intraoperative management of the hyoid bone is an important structure for mobilization during open laryngeal surgery. Total laryngectomy is the surgical management of certain advanced laryngeal cancers, particularly those that have progressed beyond the point where chemoradiotherapy alone is sufficient [8]. During the procedure, the upper boundary of the hyoid bone is identified to facilitate the detachment of the suprahyoid musculature. The hyoid bone also serves as a point of reference to identify and preserve hypoglossal nerve. Potential anatomical variations in this bone should be appreciated prior to most surgical approaches to the larynx, as was encountered in this case.

## 2. Case Report

**Figure 1:**
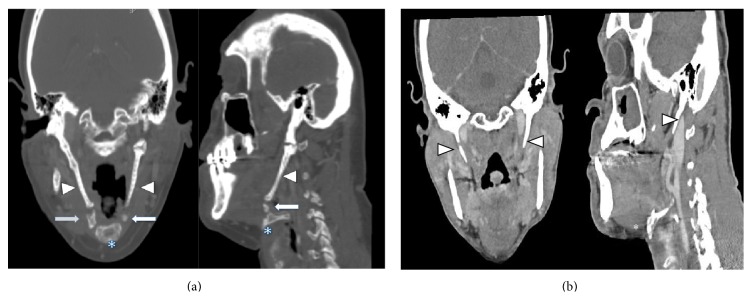
(a) CT scan of hyoid bone-styloid process extension. CT scan of hyoid bone and styloid process in patient. Arrowhead points to styloid process. Asterisk marks hyoid bone body. Thick arrow points to area of extension between hyoid bone and styloid process. (b) Normal CT scan of styloid process and hyoid bone. Normal CT scan of hyoid bone and styloid process for comparison. Arrowhead points to styloid process. Asterisk marks hyoid bone body.

The patient is a 52-year-old gentleman with no previous history of local head and neck surgery, who presented with laryngeal squamous cell carcinoma. He denied any distant history of dysphagia prior to the workup for the laryngeal cancer. External palpation of the laryngeal complex showed limited mobility. Elevation of the laryngeal complex was limited on voluntary swallowing. Modified barium swallow test showed no evidence of aspiration. Concurrent chemotherapy and radiation were originally planned, but the patient was lost to follow-up until he presented to the hospital with respiratory distress, requiring emergent surgical airway intervention. CT scan showed the presence of a large mass measuring 5.2 by 3.4 cm involving the epiglottis on the right, as well as the hypopharynx, causing displacement of the airway. An additional 3.7 by 2.3 cm the right neck mass, determined to be a metastatic lymph node, was found with significant enlargement of the sternocleidomastoid (Figure 1). He was staged as T4N2bM0; the patient consented to pursue initial surgical management of his disease to include total laryngectomy, bilateral neck dissections, and free flap reconstruction, followed by radiation.

During the case, after completion of bilateral neck dissections, the total laryngectomy was initiated. Upon identification of the hyoid bone it was noted that the greater cornu was free but the bony lesser cornu was contiguous superiorly to the styloid processes bilaterally. This intraoperative finding confirmed preoperative CT scan (Figure 1). Small areas appearing to represent discontinuity in the lesser cornu-styloid processes on CT appeared as contiguous fibrocalcified tissue without any dehiscence, strongly indicative of bony structure intraoperatively. Additionally, although the CT showed potential articulation of the elongated bony piece near the lesser cornu and temporal bone, this bony tissue was fixed to the temporal bone and the lesser cornu intraoperatively on firm digital manipulation. The lesser cornu was traced as far posteriorly as possible and then cut to facilitate removal of the suprahyoid musculature and to the styloid process was truncated to prevent postoperative dysphagia. This lesser cornu that was contiguous to the styloid process was separated after surrounding neurovascular structures were mobilized from the styloid process. Remnant styloid bone was shaved to avoid inflammation of the pharyngeal musculature. Separation of the lesser cornu from the styloid processes permitted free mobilization of the larynx, allowing for its removal with no further complications. After the completion of radiation, the patient did not complain of odynophagia or dysphagia.

## 3. Discussion

The prevailing developmental hypothesis is that the second pharyngeal arch gives rise to the stapes, styloid process of the temporal bone, stylohyoid ligament, lesser horn of the hyoid, and upper body of the hyoid, while the third pharyngeal arch gives rise to the greater horn of the hyoid and lower portion of the hyoid body (Figure 1). This dual origin theory has been supported in embryological studies involving differential inhibition of the ventral and dorsal portions of the hyoid bone [3, 9]. However, there is some uncertainty regarding the contributions of the second pharyngeal arch. Reichert's cartilage has been postulated to develop into two fragments that develop into the styloid process and a caudal segment that attaches to the lateral portion of the hyoid bone to become the lesser cornu. However, the body of the hyoid bone itself has recently been hypothesized to develop from a midline condensation of mesenchymal cells that is disparate from the second or third pharyngeal arches [3].

An alternate hypothesis argues that the hyoid bone and all of its parts originated from the third pharyngeal arch [5]. This hypothesis questions whether or not the second pharyngeal arch plays any role in development. The clear separation between the thyroid cartilage and the hyoid body in fetal development, evidenced through the presence of loose tissue between the two structures and in contrast to the tight connection between the greater horn of the hyoid and the superior cornu of the thyroid cartilage, casts doubt on the dual origin hypothesis by the implication that there is no true fusion between the arches [3].

**Figure 2:**
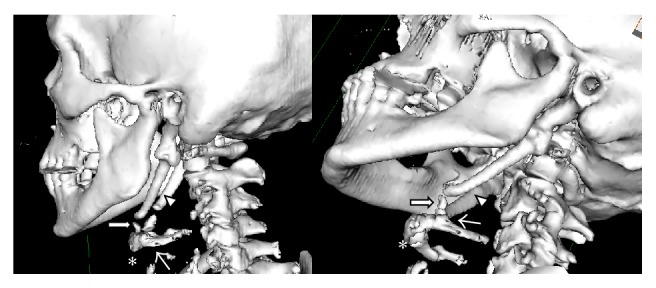
3D reconstruction of hyoid bone-styloid process extension. 3D reconstruction of hyoid bone and styloid process. Thick arrow points to area of extension between hyoid bone and styloid process. Asterisk marks hyoid bone body. Thin arrow points to lesser horn of hyoid bone. Arrowhead marks styloid process.

To our knowledge, continuation of the lesser horn of the hyoid to the styloid process has not been well documented. Isolated cases of styloid chain ossification, however, do exist in the literature and support the idea that this malformation may have developed as a consequence of an embryological defect in separation [10].  Figure 2 demonstrates that the connection between the hyoid and styloid process is a continuation of bone, rather than a calcification, in this patient. The clinical entity of styloid ligament calcification has been described before, but continuous bony structures that extend from the styloid process suggest that this is not a postdevelopmental ossification that is associated with aging [11].  Figure 1(b) demonstrates a normal CT scan for comparison. This unique anatomical case lends credence to the dual origin hypothesis, as it implies that the lesser cornu and the styloid process are derived from the same pharyngeal arch. In this case, it is possible that the extension of the caudal segment of Reichert's cartilage to the hyoid body, normally thought to form the lesser cornu of the hyoid bone, did not separate from the styloid process during development for this patient.

Other anatomic variants of the hyoid bone have been previously described in the literature. While some changes, such as the ossification of the hyoid body and greater cornu, have been associated with aging, unique anatomical variation in the hyoid bone suggests the existence of embryological malformations [12]. Some previously described cases, though rare, include bilateral absence of the lesser cornua, abnormal unilateral bone attachments to the corpus, thyroglossal cysts trapped within the hyoid bone, and abnormal or partial ossifications of the greater and lesser horns with components of the hyoid bone and surrounding structures [6, 7].

One interesting component of this styloid/hyoid abnormality is the lack of significant dysphagia in this patient prior to the onset of the laryngeal cancer. Although we did not have the ability to examine the patient prior to the onset of the laryngeal cancer to assess laryngeal complex mobility independently, our assessment on his first presentation showed limited laryngeal complex mobility but no evidence of aspiration. As such, he was initially a candidate for chemoradiation therapy. Despite this limited laryngeal complex mobility, the patient did not suffer from symptomatic dysphagia due to the near fixation of the styloid process and hyoid bone. It is unclear whether these patients would decompensate when they are challenged with other nonneoplastic laryngeal pathologies such as vocal fold mobility problems or severe laryngeal reflux. Short of any clinical symptoms, it is unclear whether prophylactic resection of these elongated styloid processes is warranted at this time. As demonstrated in this case, however, we do recommend resecting the styloid process as high as possible if surgery is performed to prevent Eagle-like syndrome. Our patient did not have any dysphagia symptoms after his surgery.

## 4. Conclusion

In reviewing this case, the presented anatomical abnormality, which may stem from an embryological malformation, increased the complexity of a total laryngectomy. While significant from a procedural standpoint, it is notable that the patient had no significant quality of life deficits as a result of this anatomical variant. The continuation of the lesser cornu and the styloid process in this patient supports the dual origin hypothesis of hyoid development and identifies a potential abnormality that may be encountered in future patients undergoing invasive head and neck surgery.
